# Fungal Communities of *Eucalyptus grandis* Leaves Are Influenced by the Insect Pest *Leptocybe invasa*

**DOI:** 10.3389/fmicb.2022.841621

**Published:** 2022-03-31

**Authors:** Mandy Messal, María Vivas, Martin Kemler, Dominik Begerow, Andreas Brachmann, Frederick Witfeld, Sanushka Naidoo, Bernard Slippers

**Affiliations:** ^1^Department of Biochemistry, Genetics and Microbiology, Forestry and Agricultural Biotechnology Institute (FABI), University of Pretoria, Pretoria, South Africa; ^2^Institute for Dehesa Research (INDEHESA), University of Extremadura, Plasencia, Spain; ^3^Evolution of Plants and Fungi, Ruhr University Bochum, Bochum, Germany; ^4^Faculty of Biology, Ludwig-Maximilians-Universität München, München, Germany

**Keywords:** *Eucalyptus* microbiome, microbial networks, phyllosphere fungal community, biotic plant stress, fungal-plant interaction, plantation trees, amplicon sequencing

## Abstract

Fungal communities in above-ground tree tissues are hyperdiverse and are influenced by biotic interactions with other organisms living in or on these tissues. These biotic interactions are, however, still poorly understood. In this study, we aimed to understand how insect-associated gall formation on *Eucalyptus* foliage correlates with the diversity of foliar fungal communities in surrounding healthy leaf tissue, as well as the co-occurrence patterns among the members of the fungal community. We used ITS metabarcoding to characterise the foliar fungal communities of 179 individual *E. grandis* trees. These trees were assigned to infestation levels of the wasp *Leptocybe invasa* (Eulophidae: Hymenoptera), which causes gall formation on shoot tips and leaves of its host. Fungal community networks were calculated using a Pearson correlation coefficient. The composition and diversity of fungal communities were influenced by the severity of *L. invasa* infestations. We identified potential *Eucalyptus* pathogens with high sequence abundance at all disease severity levels, but network analysis indicated that the co-occurrence of potential pathogens between no to mild and medium to heavy infestation differed significantly. A better understanding of microbial interactions, especially the role of pathogens, can be useful for controlling disease- and beneficial host-associated microbial communities.

## Introduction

Plant galls are the growth of abnormal plant tissue induced by other organisms (e.g. viruses, bacteria, fungi, nematodes or insects) and are found on many plant species. Insect-induced plant galls impact plant development by triggering morphological and physiological changes in the host plant tissues. Oviposition in leaf tissues initiates these cellular modifications through changes in plant development pathways, nutrient concentrations, the disruption of plant defence, selection for gall induction traits and the advent of insect-derived effectors (Giron et al., 2016; Oates et al., 2021). Plant tissues stressed in such a way, potentially also change the conditions for the colonisation or proliferation of co-existing organisms, including foliar fungi.

It is well known that fungal community diversity and the colonisation of plant host niches are influenced by abiotic, microbial and host factors (e.g. Bálint et al., 2013; Kemen, 2014; Vivas et al., 2017; Gomes et al., 2018). A clear difference between the fungal communities of healthy and yellowing *Citrus limon* leaves, where yellowing leaves had the least species diversity, exemplifies the influence of plant physiology on microbial community patterns (Douanla-Meli et al., 2013). It must therefore be assumed that galls, which function as metabolic sinks (Allison and Schultz, 2005; Dardeau et al., 2014), also influence fungal community diversity. Some studies indeed show that fungal communities in galls are different than in the surrounding leaf tissue. Cultivated fungal communities associated with aphid-induced galls in cottonwood (*Populus deltoides*) for instance exhibit distinct fungal richness and diversity in galls compared to the surrounding tissues (Lawson et al., 2014). A metabarcoding study of fungal communities showed differences in richness, diversity and composition between galls induced by *Dryocosmus kuriphilus* and surrounding chestnut leaf tissues (Fernandez-Conradi et al., 2019). Whether different levels of severity of gall formation affect the fungal community in surrounding healthy tissue is, however, not known.

*Eucalyptus* plantations are of high economic value globally (Wingfield et al., 2015). In recent years, their yield has been jeopardised by the gall-forming wasp *Leptocybe invasa* Fisher and LaSalle (Hymenoptera: Eulophidae), which deposits eggs into new growth of *Eucalyptus* trees (Naidoo et al., 2011; Dittrich-Schröder et al., 2018; Mhoswa et al., 2020). A high density of *L*. *invasa* can cause heavy galling, malformation, stunted growth and in extreme cases, tree death (Mendel et al., 2004; Zheng et al., 2014; Csóka et al., 2017). The egg oviposition into the *Eucalyptus* spp. leaf tissues initiates the expression of pathogen-related genes by the host and localised cell death causing desiccation, detachment or is directly ovicidal (Geuss et al., 2017; Griese et al., 2017). Within 24 h after oviposition, *Eucalyptus* spp. tissues accumulate reactive oxygen species and phenolics, as well as phytohormones (especially jasmonic acid, salicylic acid and ethylene) as a defence against biotic stress (Berens et al., 2017). The egg and oviposition fluid may redirect the hosts’ responses towards gall development (e.g. cell division) and is thus responsible for initiating galling (Oates et al., 2021). Gall-forming insects are also known to modify the availability of sugars, lipids and proteins in the nutritive tissue of the gall chamber (Huang et al., 2014; Ferreira et al., 2015). A study on the influence of *L. invasa* gall development on frost resistance in eucalypts found that the physiological changes on the plant foliage increased plant defence mechanisms against cold. The toll of galling by herbivores may thus have a positive indirect effect on the host plant (Rocha et al., 2013).

Fungal diversity associated with healthy and diseased *Eucalyptus* spp. has been explored for several decades (e.g. Bird et al., 1974; Bettucci and Alonso, 1997; Barbed et al., 2003; Roux et al., 2003; Hunter et al., 2011; Márquez et al., 2011; Jimu et al., 2015). The recent application of high-throughput sequencing of fungal-specific PCR amplicons has revealed enormous species diversity and richness in *Eucalyptus* spp. (Kemler et al., 2013). Such studies have identified potential pathogens existing in the fungal community of trees without visible symptoms of disease or decay. Additionally, community patterns have been shown to be highly dependent on environmental factors, as well as maternal effects that could influence the formation of fungal communities in seedlings (Vivas et al., 2017). With increasing *L. invasa* infestations in plantations (Hurley et al., 2016; Dittrich-Schröder et al., 2018), it is important to understand its influence on the associated fungal community and whether the added stress increases the occurrence of potentially pathogenic fungi.

In this study, we analysed fungal community diversity, composition and co-occurrence network structures in *E. grandis* trees with different levels of *L. invasa* infestation. We aimed to answer three questions: (i) Do *L. invasa* infestation levels correlate with fungal community diversity and composition in surrounding healthy leaf tissue?; (ii) Do co-occurrence patterns of fungal taxa correlate with *L. invasa* infestation?; and (iii) Do increased *L. invasa* infestation levels lead to an increased abundance of potential *Eucalyptus* pathogens?

## Materials and Methods

### Plant Material

A *Eucalyptus grandis* half-sib population, situated at a non-irrigated coastal site (Siya Qubeka) in KwaZulu-Natal, South Africa, was selected. The 14-month-old *Eucalyptus* trees were scored for *L. invasa* infestation symptoms. Tree infestation was characterised by one of the following categories: 0 - no infestation, 1 - infestation with evidence of oviposition, but no gall development, 2 - infestation with galls on leaves, mid-ribs or petioles and 3 - infestation with stunted overall growth and lethal gall formation. The foliar fungal communities of 179 individual trees (0, *n* = 49; 1, *n* = 50; 2, *n* = 56; 3, *n* = 24) were analysed. The *Eucalyptus* population was analysed and genotyped in previous studies (Naidoo et al., 2018; Mhoswa et al., 2020) and DNA extracts from those studies were used. Importantly, DNA was extracted from non-surface sterilised leaf punches of non-symptomatic leaf tissues and care was taken to avoid the galled areas of the leaves; thus, non-symptomatic tissue was used for all DNA samples.

### Molecular Methods and Sequencing

Polymerase Chain Reactions (PCR) were performed on the 179 *E. grandis* leaf DNA samples and PCR products were quantified through gel electrophoresis and ImageJ (Schneider et al., 2012). The primers ITS1-F (5′-CTTGGTCATTTAGAGGAAGTAA, Gardes and Bruns, 1993) and ITS4 (5’-TCCTCCGCTTATTGATATGC, White et al., 1990) were used to amplify the fungal Internal Transcribed Spacer (ITS) rDNA gene region. The amplicon library for sequencing was prepared in two consecutive PCR steps using GoTaq G2 Hot Start polymerase (Promega, Mannheim, Germany). The first PCR amplified the fungal ITS region with specific ITS1-F and ITS4 primers including a tag sequence (Supplementary Table 1). The PCR products were purified using ExoSAP-Clean Up (New England BioLabs^®^) following the manufactures’ instructions. The second PCR was conducted with primers containing the Illumina adaptor sequences to receive a unique tag-index combination (Supplementary Table 2). The products of the second PCR were quantified using 1% agarose gel electrophoresis and residual reaction chemicals were removed using the CleanPCR kit (CleanNA). Subsequently, equimolar sample pools were generated in repetitive steps. Equimolar pooled sequencing libraries (2 × 250 bp paired-end) were sequenced on an Illumina MiSeq (Illumina Inc., San Diego, CA, United States) using the MiSeq^®^ Reagent Kit v3 Chemistry at the Genomics Service Unit (LMU Biocenter, Planegg-Martinsried, Germany).

### Illumina MiSeq Sequence Processing

Sequence information was obtained as fastq files for the forward and reverse sequence reads, respectively. Samples were demultiplexed in QIIME v1.9.1 (Caporaso et al., 2010) based on forward and reverse reads. However, only the forward read was used for downstream analyses, as read lengths of 250 bp can prevent merging forward and reverse reads, thereby excluding diversity from the analyses. Subsequent sequence quality control, OTU clustering and taxonomic assignments were performed in QIIME v1.9.1 and programmes implemented therein. During quality control, reads smaller than 200 bp, containing homopolymers of a length more than 6 and a Phred score below 30, were filtered out. Chimeric sequences were removed de-novo using usearch61 (Edgar, 2010). OTUs were clustered at a 97% sequence similarity using uclust v1.2.22q (Edgar, 2010). For the taxonomic assignment, representative sequences for each OTU were queried against the UNITE database (v7_99_s_28.06.2017; Kõljalg et al., 2013) using BLAST (Altschul et al., 1990) at an e-value of 1e^−30^. For subsequent analyses, OTUs with less than ten sequences, as well as OTUs with no blast hits and non-fungal hits, were removed from the data set.

### Fungal Community Diversity and Composition

To analyse the fungal diversity among *E. grandis* trees with different *L. invasa* infestation levels, Shannon, Simpson and Invsimpson diversity indices were calculated for each tree. The effect of the different *L. invasa* infestation levels on each diversity index was analysed using a one-way ANOVA followed by a Tukey’s Honest Significant Difference (HSD) *post-hoc* test to do pairwise comparisons of the means. In all cases, model validity was checked and the *agricolae* package of the R software was used to analyse fungal diversity (R Core Team, 2018; de Mendiburu and Yaseen, 2021).

The fungal taxonomic composition of *E. grandis* leaves was summarised in a taxonomic tree using the *MetacodeR* package of the R software (Foster et al., 2017; R Core Team, 2018). Differences among community composition of *E. grandis* trees with different *L. invasa* infestation levels were visualised using a Principal Coordinate Analysis (PCoA). PCoA was performed on an abundance matrix applying Bray-Curtis dissimilarity. To assess whether community composition statistically varied among different *L. invasa* infestation levels, we employed a permutational multivariate analysis of variance (PERMANOVA). The *vegan* package of the R software (Oksanen et al., 2018; R Core Team, 2018) was used to analyse the community composition.

The community composition analysis was paired with a differential heat tree illustrating the differences in the fungal abundance of each taxon for the order Capnodiales among the *L. invasa* infestation levels using *MetacodeR*. The order of Capnodiales was selected due to their apparent role as foliar pathogens of *Eucalyptus* spp. A Wilcoxon Rank Sum test was applied, and the resulting *p*-values were corrected for multiple comparisons using the false discovery rate (FDR).

### Fungal Community Networks

Networks were calculated using the Calypso web-server (Zakrzewski et al., 2016). For this analysis, only OTUs that were assigned at least to the genus level were used. During upload, taxa that had less than 0.01% relative abundance were removed from the OTU table. Total sum normalisation and square-root transformation were performed on the dataset. Networks were calculated using a Pearson correlation coefficient and only edges that correlated significantly at *p* < 0.05 were connected. The resulting network was imported into Cytoscape v3.7.2 (Shannon et al., 2003) for depiction. To analyse the number of edges between the subnetworks, we used a one-way ANOVA followed by a Tukey’s Honest Significant Difference (HSD) *post-hoc* test to do pairwise comparisons of the means. Model validity was checked and the *agricolae* package of the R software was used to analyse fungal diversity (R Core Team, 2018; de Mendiburu and Yaseen, 2021). In order to understand possible differences in the ecology of the fungi in the network, the feeding modes of fungal genera were analysed using FungalTraits (Põlme et al., 2020).

## Results

### Sequencing

**Figure 1:**
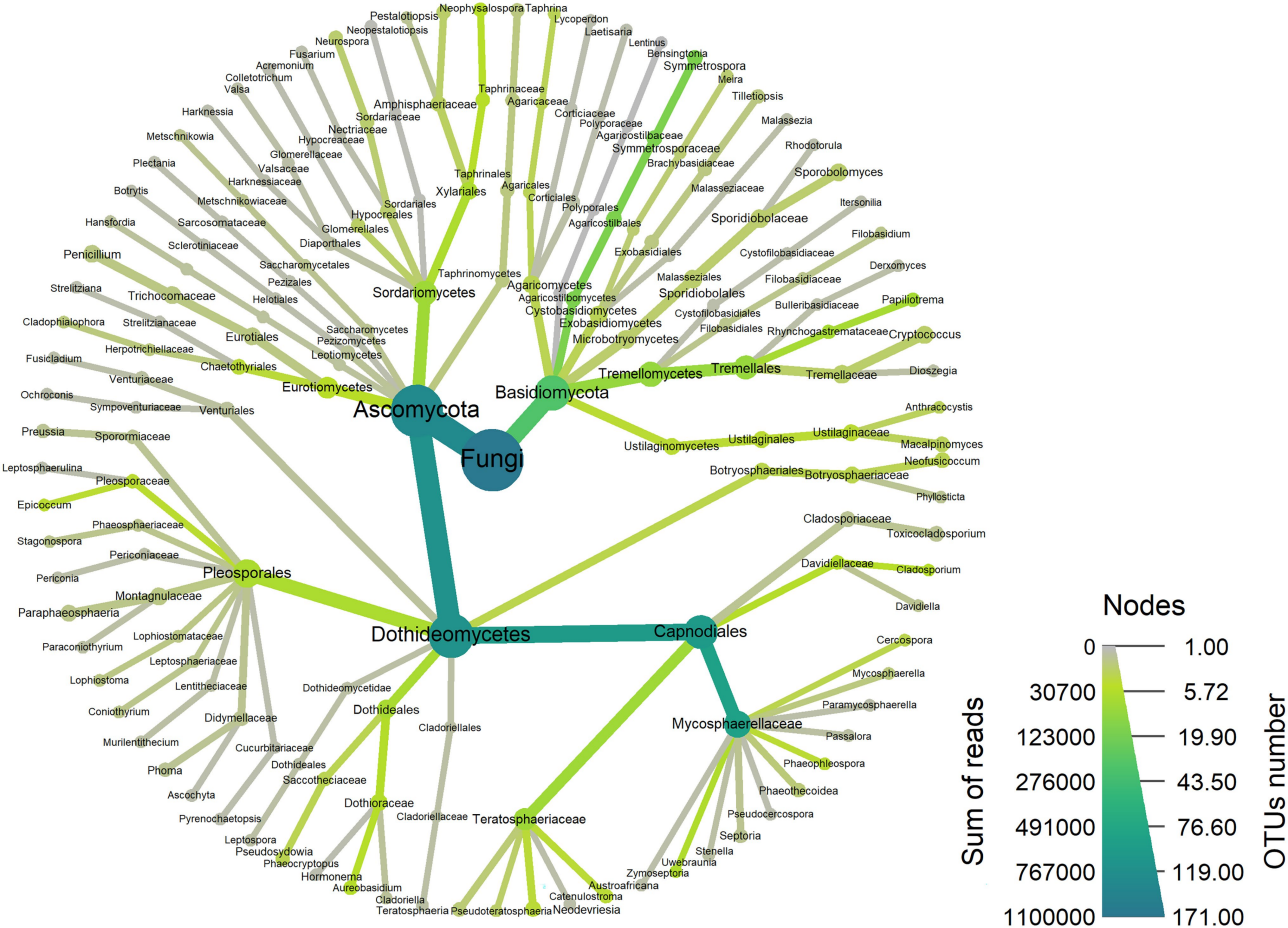
Taxonomic composition of the fungi associated with *Eucalyptus grandis* leaves. The heat tree represents the fungal community structure as a taxonomic hierarchy up to the genus level. Node and edge sizes are proportional to the number of OTUs within each taxon and colours represent absolute taxon abundance (sum of reads number).

**Figure 2:**
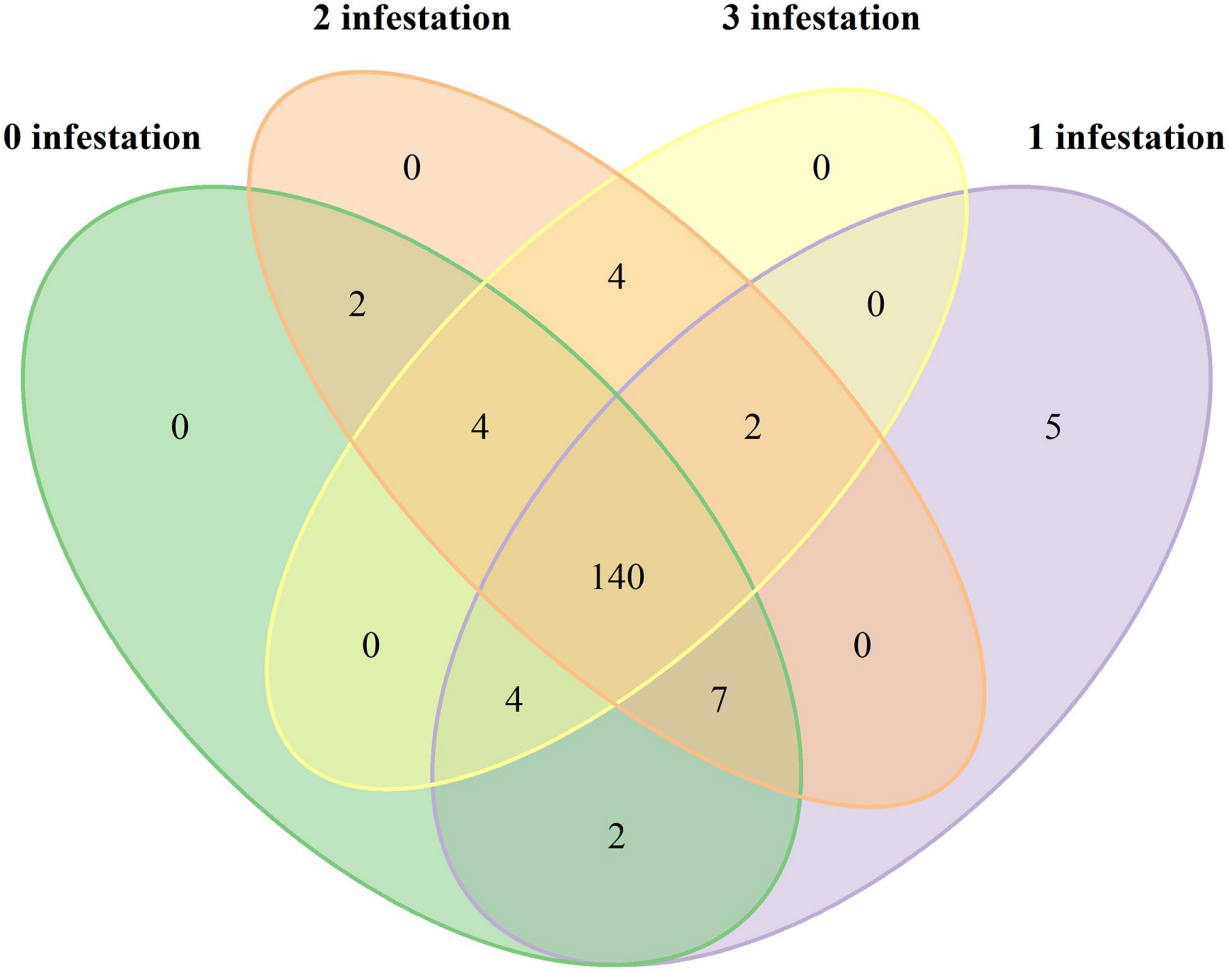
Venn diagram showing the abundance of fungal species associated with *Eucalyptus grandis* leaves among four infestation levels of the wasp *Leptocybe invasa* (0—no infestation, 1—infestation with evidence of oviposition, but no gall development, 2—infestation with galls on leaves, mid-ribs or petioles and 3—infestation with stunted overall growth and lethal gall formation).

A total of 1,104,065 filtered and non-chimeric fungal ITS1 sequences were used for community analyses. Sequencing of leaves with *L. invasa* infestation level 0 resulted in 250,261 reads, *L. invasa* infestation level 1 in 269,086 reads, *L. invasa* infestation level 2 in 377,422 reads and *L. invasa* infestation level 3 in 207,296 reads. We assigned 171 individual OTUs to 77 different fungal genera (Figure 1) and 91 different fungal species (Supplementary Tables 3–10). Out of the 171 detected OTUs, 140 OTUs were shared between the fungal communities of the four *L. invasa* infestation levels (Figure 2). We found 155 OTUs in *L. invasa* infestation level 0, 160 OTUs in *L. invasa* infestation level 1, 159 OTUs in *L. invasa* infestation level 2 and 154 OTUs in *L. invasa* infestation level 3, respectively.

### Fungal Community Diversity and Composition

**Figure 3:**
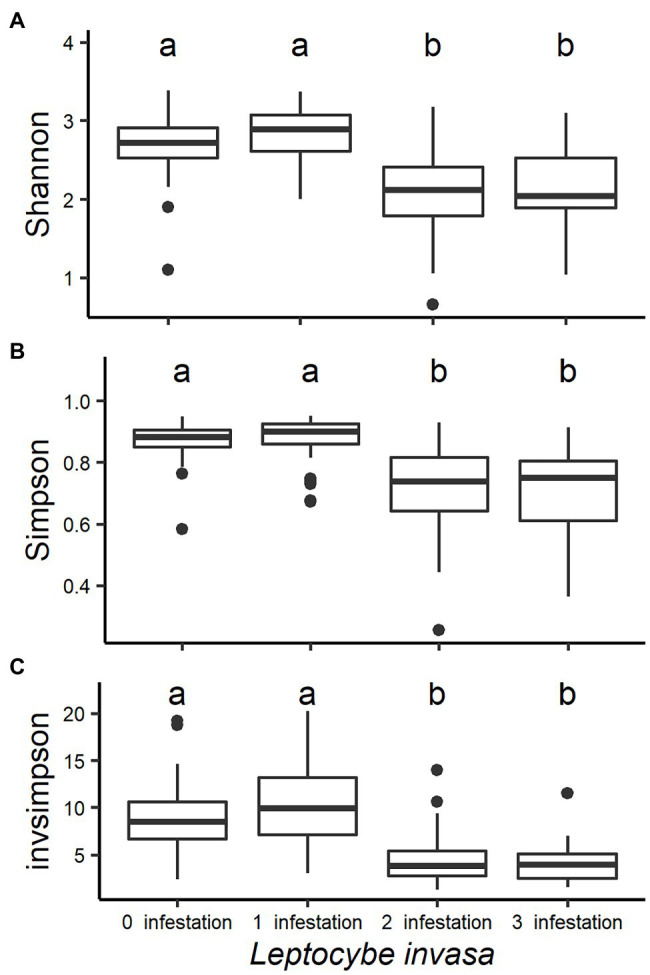
Box plots analysing the fungal diversity indices (A-Shannon, B-Simpson and C-Invsimpson) of *Eucalyptus grandis* leaves among four infestation levels of the wasp *Leptocybe invasa* (0—no infestation, 1—infestation with evidence of oviposition, but no gall development, 2—infestation with galls on leaves, mid-ribs or petioles and 3—infestation with stunted overall growth and lethal gall formation). Bars show standard errors and different letters indicate significant differences (*p* < 0.05).

Fungal community diversity was different between the four *L. invasa* infestation levels. Shannon, Simpson and Invsimpson indexes were higher in trees with low *L. invasa* infestation than in trees with a higher infestation (*p* < 0.001; Figure 3).

**Figure 4:**
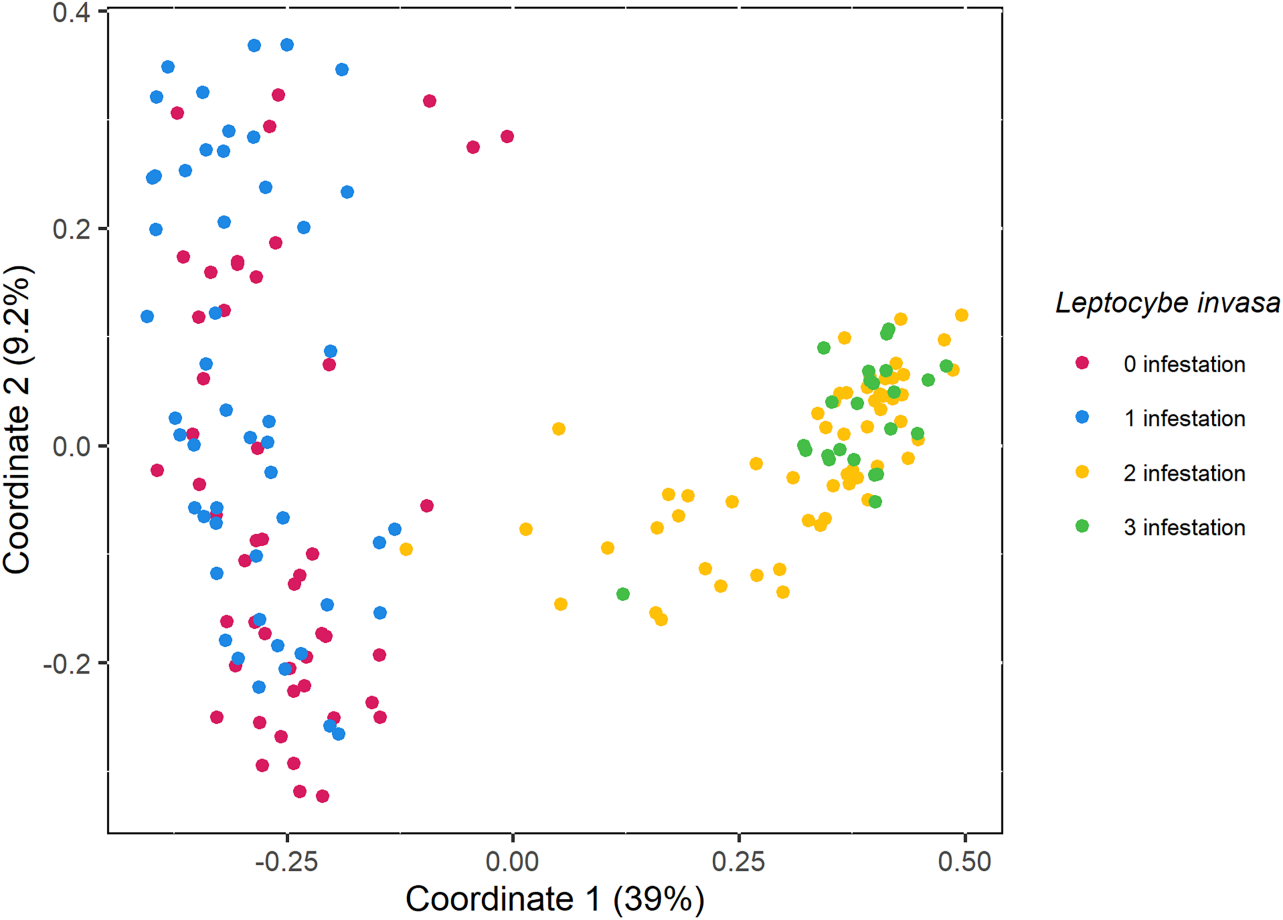
Principal coordinates analysis (PCoA) of fungal community composition associated with *Eucalyptus grandis* leaves among four infestation levels of the wasp *Leptocybe invasa* (0—no infestation, 1—infestation with evidence of oviposition, but no gall development, 2—infestation with galls on leaves, mid-ribs or petioles and 3—infestation with stunted overall growth and lethal gall formation.). The variance explained by each dimension is shown between brackets.

The PCoA plot showed that the fungal community composition differed between the *L. invasa* infestations levels 0 and 1 (healthy to mild infestation) and levels 2 and 3 (medium to high infestation), respectively (Figure 4). PERMANOVA confirmed that the *L. invasa* infestation levels significantly explained the variation in fungal community composition (*F*_1,3_ = 37.82, *r*^2^ = 0.393, *p* < 0.001).

Out of all OTUs, 67% belonged to Ascomycota, 25% to Basidiomycota and 9% could not be identified beyond Fungi. The total numbers of reads assigned to the order Capnodiales were 37% at *L. invasa* infestation level 0, 53% at level 1, 68% at level 2 and 72% at level 3. Of the total number of reads, 28% were assigned to the family Mycosphaerellaceae at *L. invasa* infestation level 0, 40% at level 1, 63% at level 2 and 69% at level 3.

**Figure 5:**
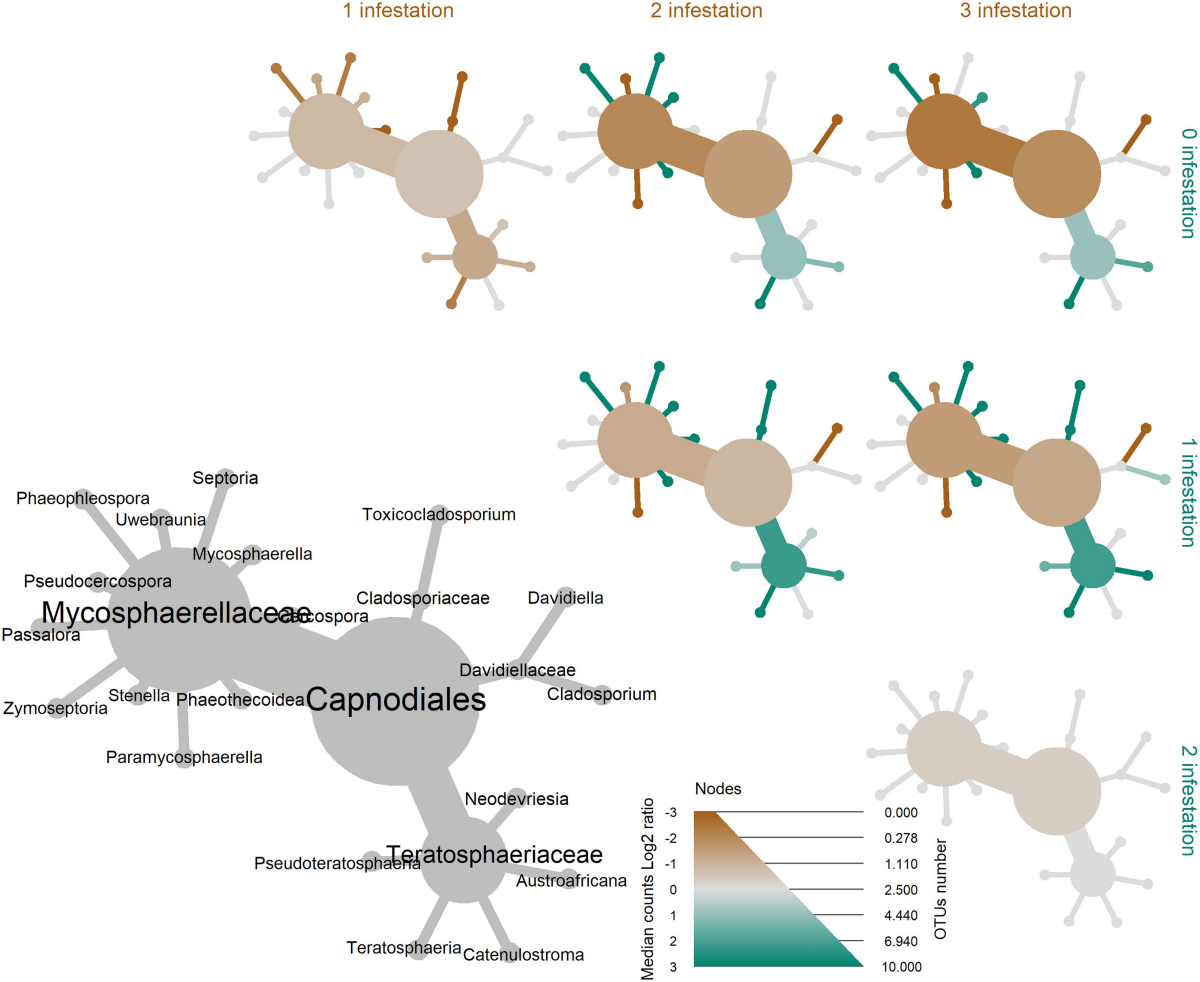
Differential heat tree up to the order level illustrating the effect of four *Leptocybe invasa* infestation levels (0—no infestation, 1—infestation with evidence of oviposition, but no gall development, 2—infestation with galls on leaves, mid-ribs or petioles and 3—infestation with stunted overall growth and lethal gall formation) on the abundance of leaf-associated fungi in *Eucalyptus grandis*. Node and edge sizes are proportional to the different number of OTUs between the groups for each taxon and colours represent the log of the ratio of median abundance between the groups for each taxon. When a taxon has more counts in samples from groups on the right side of the graph, it is coloured green. And, when a taxon has more counts in samples from groups on the upper side of the graph, it is coloured brown.

In the differential heat tree of Capnodiales, *Eucalyptus* leaves with higher *L. invasa* infestation levels showed a higher abundance of taxa in the Mycosphaerellaceae in comparison with leaves with a lower infestation level (Figure 5). Most of the OTUs within the Capnodiales (22 out of 37 OTUs) were assigned to the family Mycosphaerellaceae.

### Fungal Community Network Results

**Figure 6:**
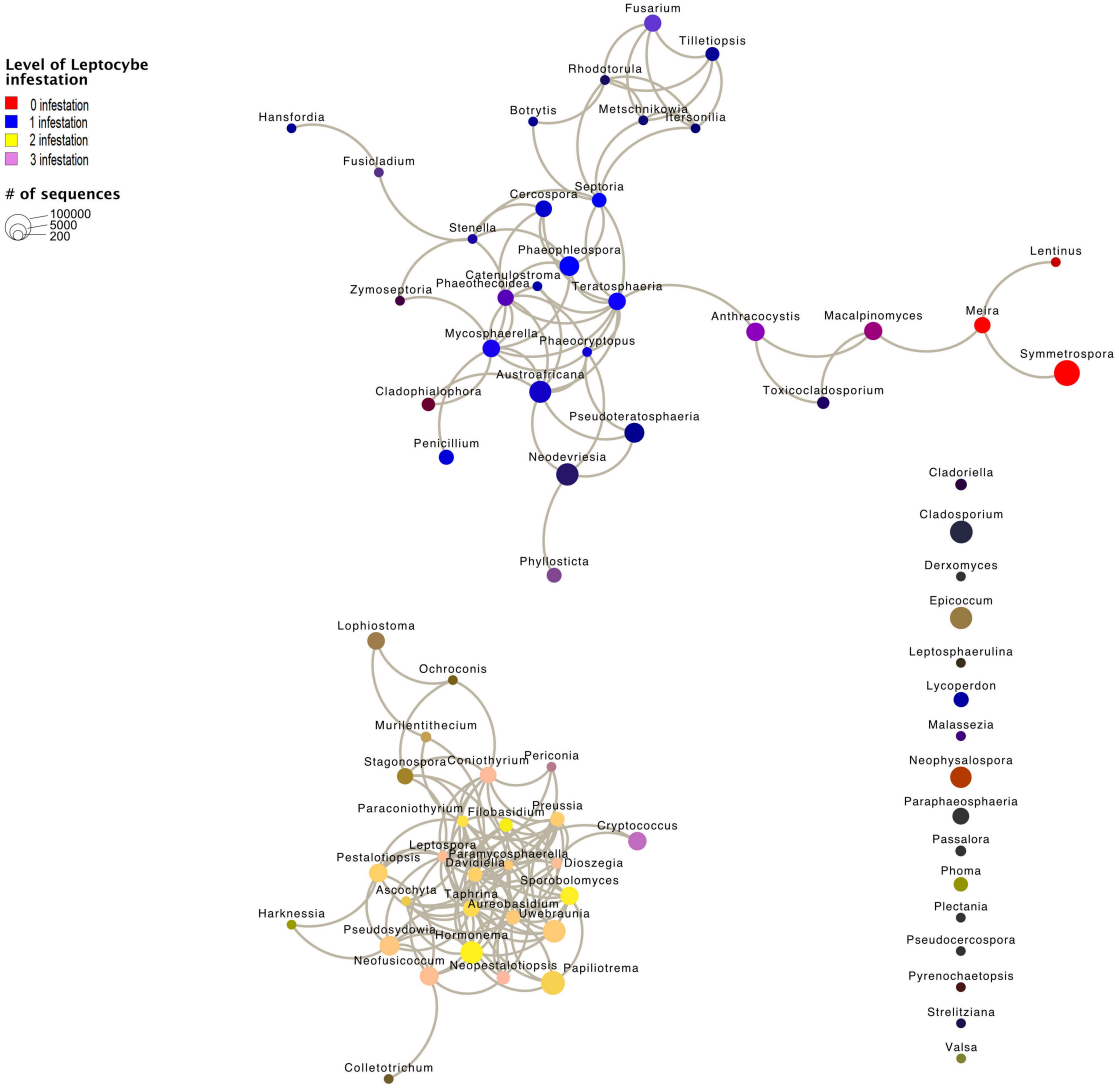
Network graph calculated using Calypso. Node size represents the abundance of the genus. Edges are significant correlations between the nodes. Node colour is made up of four infestation levels of the wasp *Leptocybe invasa* (0—[red] no infestation, 1—[blue] infestation with evidence of oviposition, but no gall development, 2—[yellow] infestation with galls on leaves, mid-ribs or petioles and 3—[mauve] infestation with stunted overall growth and lethal gall formation). Node colours are merged based on the correlation to the *Leptocybe invasa* infestation level, e.g. *Fusarium* (purple) appears mainly in *Leptocybe invasa* infestation levels 0 and 1 samples. 16 fungal genera did not show significant correlations.

We recovered two subnetworks that did not show any statistically significant co-occurrence among each other and 16 genera that did not show any significant co-occurrence patterns (Figure 6). Subnetwork 1 represents genera, which were largely retrieved from samples with *L. invasa* infestation levels 0 or 1 (red and blue in Figure 6) and subnetwork 2 represents genera, which were largely retrieved from samples with *L. invasa* infestation levels 2 or 3 (yellow and mauve in Figure 6). Fungal genera occurring in subnetwork1 have significant fewer edges (61) than fungal taxa in subnetwork 2 (98; *p* < 0.001, Table 1; Supplementary Figure 1; Supplementary Table 11), while the number of nodes is comparable (subnetwork 1 = 30, subnetwork 2 = 26). In our network analysis, a node represents a taxon or OTU grouped at a specific level, e.g. genus level, while edges are lines connecting nodes and represent significant correlations between these nodes.

**Table 1:** Summary network statistics calculated in Cytoscape.

	Subnetwork 1	Subnetwork 2
Number of nodes	30	26
Number of edges	61	98
Average number of neighbours	4.067	7.538

The subnetworks refer to Figure 6 where subnetwork 1 is made up of fungal genera with high abundances in samples with *Leptocybe invasa* infestation levels 0 and 1 and subnetwork 2 is made up of fungal genera with high abundances in samples with *Leptocybe invasa* infestation levels 2 and 3.

The fungal feeding modes were analysed using FungalTraits (Põlme et al., 2020). No difference could be observed between feeding types of the fungal genera of subnetwork 1 (*L. invasa* infestation level 0 and 1) compared to subnetwork 2 (*L. invasa* infestation level 0 and 1). In both subnetworks, the majority of genera were classified as plant pathogens (Supplementary Table 10; Supplementary Figure 2).

## Discussion

In this study, we show that the fungal community structure of *E. grandis* leaves is influenced by the insect pest *L. invasa*. We characterised a high fungal diversity, with 171 fungal taxa identified from these leaf tissues in one site. The fungal communities were clearly structured by the level of *L. invasa* infestation. Potential phytopathogenic taxa were present in all four levels of infested leaves. This is the first demonstration of such a clear relationship between the level of *L. invasa* infestation in *E. grandis* leaves and fungal community structure.

Leaves with no or mild *L. invasa* infestation (*L. invasa* infestation levels 0 and 1) had a significantly higher fungal community diversity than leaves showing medium and high infestation (*L. invasa* infestation levels 2 and 3). It has been shown before that a plant’s health status influences its associated fungal diversity. For example, gall tissue induced by the gall wasp *Dryocosmus kuriphilus* in chestnut leaves harboured a significantly lower fungal community diversity compared to the surrounding plant tissue (Fernandez-Conradi et al., 2019) and increasing levels of powdery mildew infection in pumpkin leaves correlated negatively with fungal community diversity (Zhang et al., 2018). The correlations between increased infection levels and fungal community diversity are, however, controversial. While *Fusarium* head blight infections increase *Fusarium* spp. in wheat spikelets and kernels, overall fungal community diversity only reduced significantly in kernels and not in spikelets (Rojas et al., 2020).

In this study, we sampled healthy tissues that were adjacent to the affected tissues. This indicated that galling has indirect effects on fungal species diversity in non-symptomatic tissues. Changes in fungal community diversity have been attributed to factors including changes in plant nutrients, secondary metabolites, competition, plant defence reactions, as well as phytohormone regulation (Cameron et al., 2013; Pieterse et al., 2014; Abdelfattah et al., 2016; Bennett and Cahill, 2016; Shen et al., 2018; Gluck-Thaler et al., 2020). These changes can also be seen in the surrounding gall tissues, hence affecting the host’s mycobiome (Lawson et al., 2014; Fernandez-Conradi et al., 2019).

The *L. invasa* infestation did not only lower fungal diversity within respective leaves but also led to a deterministic pattern of fungal community composition between leaves of different infestation levels. The communities associated with no or mild *L. invasa* infestation and those associated with a medium or heavy infestation were clearly separated on PCo 1. Interestingly, there was no gradual transition between infestation levels, but two separate clusters were formed of leaves with no/mild infestation and leaves with medium/heavy infestation. This could indicate a limitation in our scoring system, as we might have been unable to determine subtle infections in what were assumed to be healthy leaves. However, the clear separation between two clusters might also be consistent with an interpretation that the *Leptocybe*-*Eucalyptus* interaction causes a threshold, where the associated fungal community switches in composition. Such tipping points have been observed in other host-associated microbiomes (Tytgat et al., 2019) and raise interesting questions about the conditions at which such bifurcations occur in the tripartite interaction of host, *Leptocybe* and mycobiome for further investigation.

Fungal communities on healthy or mildly infested leaves (*L. invasa* infestation levels 0 and 1) showed more dispersed fungal community composition than heavily and medium infested leaves (*L. invasa* infestation levels 2 and 3). Such differences have been observed in disease systems before, except that it was mostly the sick individuals that showed a more dispersed diversity (Turnbaugh et al., 2009; Hong et al., 2015). This so-called Anna Karenina principle has been attributed to a more stochastic distribution of factors affecting the structure of microbial communities in non-healthy individuals and predicts that all healthy microbiomes are alike, and each disease-associated microbiome is ‘sick’ in its own way (Zaneveld et al., 2017). The reason for the less dispersed composition in sick individuals, and how common this might be in plant gall associated microbial communities, is not known.

A sudden shift in *Eucalyptus* leaf physiology due to galling could explain our observed shift in fungal diversity. Putative physiological changes due to gall development and *Eucalyptus* defence mechanisms have been observed, including cell wall reinforcement, protease inhibitors, cell cycle suppression and regulatory hormone signalling pathways (Oates et al., 2015). It is well known that fungi take advantage of the physiological state of their host. For example, when comparing the fungal communities of healthy and yellowing *C. limon* leaves, a result of either nutrient deficiency or drought stress, the potential phytopathogen *Colletotrichum gloeosporioides* increased in abundance, whereas most other fungal species decreased (Douanla-Meli et al., 2013). These fungal species may have advantageous traits for coping with physiological disturbance, whereas the other fungal endophytes might rely on nutrition and protection from their healthy plant host.

Oviposition initiates physiological changes in leaf tissues, including cellular modifications, changes in plant development pathways and nutrient concentrations, the disruption of plant defence, selection for gall induction traits and the advent of insect-derived effectors (Giron et al., 2016). The cynipid gall wasp *Andricus petiolicolus*, for example elicits galls on chestnut oak leaves (*Quercus prinus*) that significantly differ from the leaf tissue from which they are formed (Allison and Schultz, 2005). The gall cortex and epidermis exhibit higher peroxidase and invertase activities and greater condensed tannin concentrations than the nutritive tissues or leaves. The woolly poplar aphid *Phloeomyzus passerinii* can induce a pseudogall within the bark on trunks of poplars, where the gall acts as a sink accumulating nutrients, like amino acids, soluble sugars or starch drawn from the surrounding tissues (Dardeau et al., 2014). A study on the transcriptome and terpene profiles of *E. grandis* challenged with *L. invasa* shows that indeed changes are induced by oviposition, including mono- and sesquiterpene profiles, phytohormone responses and lignification locally at the site of oviposition (Oates et al., 2015). A joint transcriptomics study (Dual-RNA) analysing plant and fungal expressed genes associated with insect gall development will increase the understanding of these biotic interactions.

Distinct fungal community subnetworks could be observed between healthy/mildly infested and medium/heavily infested *E. grandis* leaf samples. All but 16 genera showed significant co-occurrence within their subnetworks, i.e. nodes are connected by edges. Even though co-occurrence is not evidence of ecological interactions (Blanchet et al., 2020), we observe a non-random co-occurrence pattern, possibly caused by a biological driver. The higher number of edges in subnetwork 2 could be explained by the physiological changes of *L. invasa* gall development on the host plant, which outweigh the impact of general environmental factors. These findings might also be influenced by biotic factors, e.g. microbial interactions. Subnetwork 2 also has a higher average number of neighbours than subnetwork 1, which agrees with the fungal community composition pattern shown in our PCoA and PERMANOVA analyses.

Trait analysis suggested that potential phytopathogens occur in the fungal community of both, *L. invasa* infested tissues, as well as healthy/mildly infested plants. Fungi that are reputedly ‘phytopathogenic’ were also recovered from healthy plant tissues in multiple previous studies (Rodriguez et al., 2009; Hardoim et al., 2015; Almario et al., 2017). It has been proposed that stress (e.g. pathogen attack or drought) impacts a plant’s microbiome, likely *via* alterations in physiology, which could result in favourable conditions for pathogens to cause disease (Slippers and Wingfield, 2007; Sherwood et al., 2015; Oliva et al., 2021). Such stress is, however, not a prerequisite for infection by pathogens, who then remain dormant until conditions are conducive to their further development.

We found an increase in abundance of several Mycosphaerellaceae taxa with increasing *L. invasa* infestation level, indicating that some Mycosphaerellaceae taxa are favoured by the morphological and physiological changes in the leaf *E. grandis* environment after *L. invasa* oviposition. Our results support a newly proposed micro-evolutionary approach of fungal ecological niches (Selosse et al., 2018). We have at least two different micro-environments in our study, namely, the healthy and the gall surrounding tissues. Growing evidence that many fungi have more complex niche adaptations than previously imagined has been reported (Selosse et al., 2018). Although the number of pathogenic taxa in our study is similar between the micro-environments, some pathogenic taxa (i.e. Mycosphaerellaceae) have more counts (higher abundance) in gall surrounding tissues compared to healthy leaf tissues. Our study raises the questions, why potentially pathogenic taxa abundances change and what the relevance of microbial interactions following physiological changes in the host is Carrión et al. (2019).

In conclusion, we were able to show that the fungal communities surrounding *L. invasa* oviposition sites change with an increasing infestation level. This knowledge helps to further understand the outcomes of the *E. grandis*-*L. invasa* interaction, as well as factors that influence fungal community composition in general. Future work could use functional genetic studies to untangle the intricacies of biotic interactions in this system by using combined microscopic, transcriptomic, proteomic and metabolomic approaches.

## Data Availability Statement

The datasets presented in this study can be found in online repositories. The names of the repository/repositories and accession number(s) can be found at https://www.ncbi.nlm.nih.gov/, PRJNA791551.

## Author Contributions

BS, MK and SN conceived the study. FW and MM prepared the sequence library. AB performed Illumina sequencing. MM, MV, and MK performed the data analysis, visualisation and interpretation. MM drafted the initial manuscript. BS, SN and DB obtained funding to support the research. All authors contributed to the article and approved the submitted version.

## Funding

Funding for this work was provided by members of the Tree Protection Co-operative Programme (TPCP) and the DST/NRF Centre of Excellence in Plant Health Biotechnology (CPHB). The funders had no role in study design, data collection and analysis, decision to publish or preparation of the manuscripts.

## Conflict of Interest

The authors declare that the research was conducted in the absence of any commercial or financial relationships that could be construed as a potential conflict of interest.

## Publisher’s Note

All claims expressed in this article are solely those of the authors and do not necessarily represent those of their affiliated organizations, or those of the publisher, the editors and the reviewers. Any product that may be evaluated in this article, or claim that may be made by its manufacturer, is not guaranteed or endorsed by the publisher.
